# A Lightweight Remote Sensing Small Target Image Detection Algorithm Based on Improved YOLOv8

**DOI:** 10.3390/s24092952

**Published:** 2024-05-06

**Authors:** Haijiao Nie, Huanli Pang, Mingyang Ma, Ruikai Zheng

**Affiliations:** School of Computer Science and Engineering, Changchun University of Technology, Changchun 130012, China; 2202203071@stu.ccut.edu.cn (H.N.); 2202103032@stu.ccut.edu.cn (M.M.); 2202203089@stu.ccut.edu.cn (R.Z.)

**Keywords:** small object detection, remote sensing image, YOLOv8n, HPANet, SSFF

## Abstract

In response to the challenges posed by small objects in remote sensing images, such as low resolution, complex backgrounds, and severe occlusions, this paper proposes a lightweight improved model based on YOLOv8n. During the detection of small objects, the feature fusion part of the YOLOv8n algorithm retrieves relatively fewer features of small objects from the backbone network compared to large objects, resulting in low detection accuracy for small objects. To address this issue, firstly, this paper adds a dedicated small object detection layer in the feature fusion network to better integrate the features of small objects into the feature fusion part of the model. Secondly, the SSFF module is introduced to facilitate multi-scale feature fusion, enabling the model to capture more gradient paths and further improve accuracy while reducing model parameters. Finally, the HPANet structure is proposed, replacing the Path Aggregation Network with HPANet. Compared to the original YOLOv8n algorithm, the recognition accuracy of mAP@0.5 on the VisDrone data set and the AI-TOD data set has increased by 14.3% and 17.9%, respectively, while the recognition accuracy of mAP@0.5:0.95 has increased by 17.1% and 19.8%, respectively. The proposed method reduces the parameter count by 33% and the model size by 31.7% compared to the original model. Experimental results demonstrate that the proposed method can quickly and accurately identify small objects in complex backgrounds.

## 1. Introduction

With the rapid development of the information age, remote sensing images play an extremely important role in many fields, such as ecological resource and climate monitoring, military target detection, medical diagnosis, and Unmanned Aerial Vehicle navigation [1]. For example, in recent years, as atmospheric warming has led to the melting of glaciers and sea level rise has led to frequent disasters, such as floods and mudslides [2], the detection technology of remote sensing images can predict the occurrence of such disasters through the change in geographic features in the images, so as to save people’s lives and properties. Furthermore, remote sensing image detection technology can also be applied to active SAR [3] and passive microwave RS [4] techniques for soil moisture detection. This technology enables the rapid and accurate identification of sensitive information in the data, thereby achieving precise detection. Even in cases where satellite data resolution is low, remote sensing image technology can still enhance accuracy through data augmentation and by selecting training data and adjusting the model parameters accordingly to accomplish tasks. This illustrates the wide-ranging impact of remote sensing image detection technology in many important fields. However, small targets in remote sensing images are usually characterized by low resolution, their propensity to be easily obscured by other objects, complex and changeable backgrounds, the greater influence of light and weather conditions, and a small number of small targets with wide distribution. These characteristics have caused great difficulties in the accurate detection of objects in remote sensing images. Therefore, small target detection in remote sensing images has become an important research direction in the development of the computer vision field.

The ways of defining micro-sized targets are mainly categorized into definitions based on relative scales and definitions based on absolute scales. One kind of definition is by relative scale. When the relative area of the target instances is between 0.08% and 0.58%, they can be considered as a whole. It is also possible to go through the ratio of the width and height values of the target bounding box to the width and height values of the image to accurately represent them, or the ratio of the area of the target bounding box to the area of the image to accurately represent them. Although using this type of definition can provide some help, it also has some challenges, such as not being able to accurately assess the model’s ability to detect targets at various scales and also being susceptible to data preprocessing and model structure. Another type of definition is based on absolute scales. Based on the MS COCO data set [5] and what is known in practical applications, objects with a resolution lower than 32 pixels × 32 pixels can be called small targets. The concept of small targets can also be defined according to different situations, such as the complexity of the real environment and the complexity of the convolutional neural network. Besides MS COCO, there are other data sets that give definitions of small targets based on absolute scales. For example, in the TinyPerson data set [6], targets with pixel value sizes between [20 and 32] are defined as small targets. In the face detection data set WIDER FACE [7] and in the aerial image data set DOTA, targets with pixel values between [10 and 50] are defined as small targets.

Traditional object detection methods often rely on feature extraction to detect image information, such as Histogram of Oriented Gradients (HOG) [8], Scale-Invariant Feature Transform (SIFT) [9], Local Binary Pattern Histograms (LBPHs) [10], etc. Although these traditional methods have certain advantages in extracting local image features and designing classifiers, they suffer from limitations, such as poor robustness, weak generalization ability, high computational cost, and difficulty in solving feature representation problems. As a result, these models cannot effectively detect small targets in complex scenes in remote sensing images. In recent years, with the development of computer hardware and the improvement of computational capabilities, deep learning has become a popular research direction in the field of artificial intelligence. Among deep learning algorithms, object detection algorithms based on Convolutional Neural Networks (CNNs) have gradually replaced traditional object detection algorithms due to their weight sharing and translational invariance properties, thus achieving faster computation speed and higher detection accuracy. They have become the mainstream detection method.

Based on deep learning, object detection algorithms are mainly divided into two categories: two-stage detection and single-stage detection. Two-stage detection first extracts features from the image during the detection process and generates some candidate regions and then classifies and locates the candidate regions to output the position and category information of the targets. Typical two-stage detection algorithms include R-CNN [11], Fast R-CNN [12], and Faster R-CNN [13]. Single-stage detection uses CNN to extract image features, passes the extracted features to multiple fully connected layers for object detection, and then directly outputs the position and category information of the targets. Typical single-stage detection algorithms include the YOLO series [14,15,16,17] and SSD [18]. Single-stage detection algorithms can process and analyze information in images without generating candidate regions in advance, thus eliminating frequent data transformations and computations and thereby greatly improving detection efficiency. Therefore, in large-scale data processing, single-stage detection algorithms are more widely applied than two-stage detection algorithms.

Currently, there is still significant room for improvement in the detection accuracy of small targets in remote sensing images. The main challenges stem from the characteristics of such images, including the low resolution of small targets, susceptibility to occlusion by other objects, complex and variable backgrounds, the significant influence from lighting and weather conditions, and the sparse distribution of small targets. Additionally, downsampling in image processing may lead to loss of object information, resulting in sparse representation of object features in high-level feature maps. All of these factors collectively impact the detection of small targets in remote sensing images. To address these challenges, this paper proposes a lightweight small target detection algorithm based on YOLOv8n. Introducing a small target detection layer allows for better preservation of small target feature information. Combined with the SSFF module, it enables the model to better integrate the high-dimensional information of deep feature maps and the detailed information of shallow feature maps. Furthermore, the proposed and applied Path Aggregation Network (HPANet) strengthens the fusion of feature maps at different scales.

The main contributions of this paper are summarized as follows:

(1) A detection layer is added to the original multi-scale feature fusion network structure, thus generating larger-scale feature maps and improving the network’s ability to learn small target feature information.
(2) It incorporates the SSFF module by selectively fusing feature maps of different scales, thus improving the detection accuracy and robustness of small targets and reducing the number of model parameters.
(3) The HPANet network is proposed, and it replaces the original PANet network, thus enhancing the network’s ability to integrate feature maps of different scales while simultaneously reducing network parameters.

## 2. Related Work

Small object detection has wide applications and practical uses in various fields, and many scholars are dedicated to improving the detection accuracy of small objects. Although single-stage detection algorithms represented by the YOLO series outperform many object detection methods, remote sensing images typically have various characteristics, such as a high proportion of background information, a high degree of target clustering, and a large number of small objects. These characteristics result in lower detection accuracy of the YOLO series compared to two-stage detection algorithms. To improve the detection performance of objects in remote sensing images, many researchers have made improvements to the YOLO series. Ma et al. [19] proposed SP-YOLOv8s based on YOLOv8s, replacing the cross-row convolution module with the SPD-Conv module to enhance the network’s learning and representation capabilities. Additionally, they replaced the path aggregation network with the SPANet structure to enhance feature fusion at different scales. Wang et al. [20] introduced a C2f-E structure based on an efficient multi-scale attention module (EMA) to enhance the network’s detection capability for objects of different sizes in remote sensing images. Han et al. [21] proposed a VEW-YOLOv8n network model, where the backbone network utilized the VanillaC2f module, thus reducing the complexity and number of parameters in the model. Jang et al. [22] presented a lightweight forest pest image recognition model based on the improved YOLOv8 architecture. This algorithm replaced the traditional convolutional layers in the YOLOv8 neck module with lightweight GSConv and utilized a Slim neck design paradigm for reconstruction, thus reducing computational costs while maintaining model accuracy. Wang et al. [23] proposed a model for Unmanned Aerial Vehicle (UAV) strawberry recognition based on YOLOv8. The improved YOLOv8 model incorporates a Shuffle Attention Module and a VoV-GSCSP Module, thereby enhancing both recognition accuracy and detection speed. Ling et al. [24] introduced the YOLOv8s-GhostNetv2-CA_H model based on YOLOv8. This model replaces the backbone of the YOLOv8 model with GhostNetv2 architecture for lightweight conversion. Additionally, it integrates an improved CA_H attention mechanism, successfully achieving precise detection of jujube tree trunks. Fan et al. [25] addressed the issue of limited computing resources in coal mines by proposing the CM-YOLOv8 model. This algorithm introduces adaptive predefined anchor boxes tailored to the coal mining data set to enhance detection performance for various targets. Moreover, it devises a pruning method based on the L1 criterion, thus significantly compressing both the computational and parameter volume of the model without compromising accuracy. The experimental results demonstrate its effectiveness.

In the above researchers’ studies, it can be seen that the algorithms of the YOLO series are widely used in various application fields, with significant potential for improvement in some specific areas. This paper proposes a lightweight enhanced YOLOv8n method for remote sensing image target detection, which achieves good detection results on the VisDrone remote sensing data set.

## 3. Introduction of YOLOv8 Detection Network

YOLOv8, proposed by Ultralytics, is a deep neural network based on a single-stage object detection algorithm, thus providing a new state-of-the-art (SOTA) model. It offers models of different sizes, including N, S, M, L, and X scales, based on scaling factors to meet various scene requirements. YOLOv8 supports various visual tasks, integrating algorithms like pose estimation, object detection, image classification, and instance segmentation. It adopts multiple SOTA technologies, demonstrating strong scalability to support other YOLO versions and algorithms beyond YOLO. YOLOv8 adopts the C2f structure, with a richer gradient flow in the backbone network and neck end, combining semantic information with contextual information. It sets different channel numbers for models of different scales to improve overall performance. Its detection head adopts a decoupled head structure, thus separating detection and classification tasks and independently processing detection tasks. YOLOv8 uses binary cross-entropy as a classification loss function.

(1) $$\;H\left(y,\hat{y}\right)=-\frac{1}{N}\sum_{i=1}^{N}\left[y_{i}\log\left({\hat{y}}_{i}\right)+\left(1-y_{i}\right)\log\left(1-{\hat{y}}_{i}\right)\right]$$

Binary cross-entropy (BCE) is a commonly used loss function in computer vision, which is usually used in binary classification problems, such as the presence of an object in an image or whether the image is normal or abnormal. This function plays an important role in evaluating the performance of the model and in the training process. $H\left(y,\hat{y}\right)$ is the binary cross-entropy loss function, where y is the actual label, which is usually a vector containing 0 and 1 (if it is 0, it means a negative case, and if it is 1, it means a positive case). $\hat{y}$ is the predicted output of the model, which is also usually a vector containing 0 and 1, and it represents the probability that the model belongs to a positive case for each sample. N is the number of samples, and $y_{i}$ and ${\hat{y}}_{i}$ are the ith element of the label and the predicted output.

YOLOv8 uses DFL (distribution focal loss) and CIoU loss as regression loss functions. CIoU (Complete IoU) can more accurately measure the similarity between target frames, which improves the accuracy and stability of target detection. The DFL (Detection Fusion Layer) refers to a technology layer used for target detection. Its main purpose is to fuse the detection results from different feature layers to improve the performance of target detection. The CIoU and DFL formulas are shown below, respectively.

(2) $$\;CIOU=IoU-p\left(C_{2}\;,\;C_{1}\right)-\gamma v\left(C_{2}\;,C_{1}\right)$$

where IoU denotes the ratio of intersection to concatenation between the measured frame and the real bounding box. $p\left(C_{2}\;,\;C_{1}\right)$ is a normalized term for the distance between the centroids of the two frames. $v\left(C_{2}\;,C_{1}\right)$ is a normalized term for the distance between the predicted frame and the diagonal length of the real frame. γ is an adjustable parameter that balances the importance of the centroid distances and the diagonal lengths. ciou combines these terms in order to comprehensively assess the degree of match between the predicted box and the real bounding box. It takes into account certain aspects, such as the location, size, and shape of the box, making it more comprehensive and accurate in evaluating the performance of the target detector.

(3) $$\;{DFL}_{i,j}=\sigma \left(\alpha \cdot F_{i,j}+\beta \cdot G_{i,j}\right)$$

where ${DFL}_{i,j}$ denotes the final detection result of the ith feature layer and the jth position, $F_{i,j}$ denotes the detection result from the ith feature layer, $G_{i,j}$ denotes the detection result from the jth position, α and β are the fusion weights, and σ is the activation function sigmoid. The DFL combines the linearly weighted detection results from the different feature layers and positions and performs a nonlinear transformation through the activation function to generate the final detection result.

**Figure 1:**
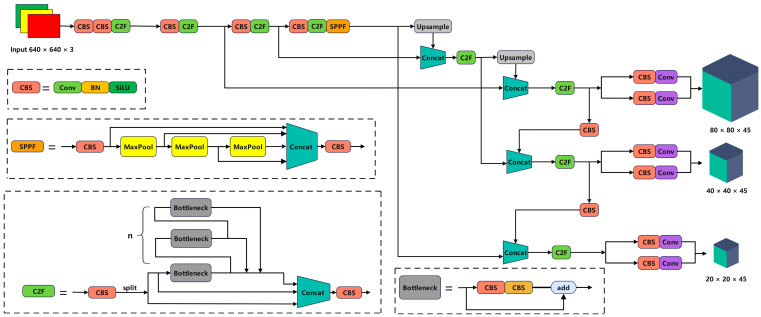
The YOLOv8 architecture.

Compared to previous versions, YOLOv8 also introduces a semantic segmentation model, YOLOv8-Seg. YOLOv8 improves the overall performance by optimizing the network structure to achieve higher and more flexible detection results suitable for a wide range of engineering applications. The model is trained on large-scale data sets, demonstrating good generalization ability, and it can be applied to various object detection tasks. In this paper, we take into account the accuracy and speed of each model in YOLOv8, and we choose the YOLOv8n network as the base model. The network structure of YOLOv8 is shown in Figure 1.

## 4. Improvement of YOLOv8

### 4.1. Small Target Feature Retention Layer

**Figure 2:**
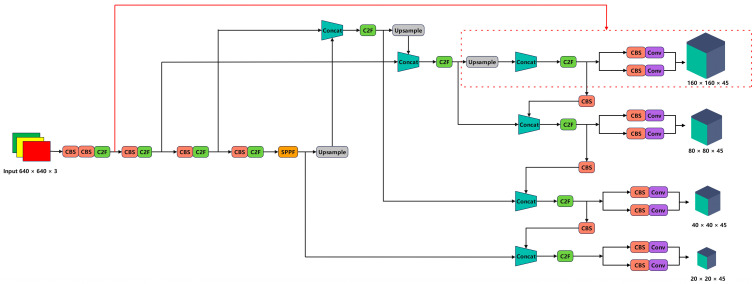
The YOLOv8 architecture with the addition of a small object detection layer.

In the original YOLOv8 network, the larger downsampling ratio resulted in smaller initial downsampling sizes of the feature maps. After multiple downsampling operations, it becomes very difficult to fully preserve the feature information of small objects. Consequently, the feature information in the neck network of YOLOv8 cannot be adequately fused, leading to decreased detection accuracy. In deep learning, smaller receptive fields allow the backbone network to obtain more fine-grained information, thereby improving the accuracy of small object detection. To address this issue, we introduced the shallow feature layer P2 outputted by the backbone network into the feature fusion network structure and made corresponding improvements. Specifically, the FPN module in the neck structure [26] generates 80 × 80 feature maps through upsampling, followed by additional upsampling operations to obtain larger 160 × 160 feature maps. These feature maps are then fused with the 160 × 160 feature maps generated in the backbone structure and inputted into the head structure for classification and detection, thus extracting feature information of small objects from shallower feature maps. These improvements enable the network to better preserve the feature information of small objects and enhance the detection accuracy of small objects through the fusion of shallow and deep feature maps. The newly added small object detection layer is illustrated in Figure 2.

### 4.2. SSFF Module

**Figure 3:**
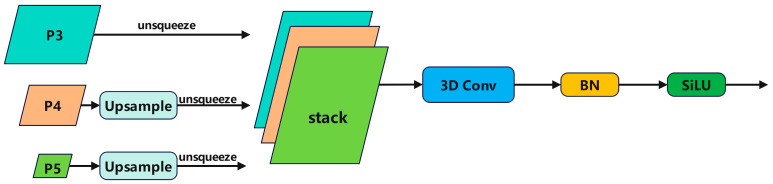
The SSFF architecture.

For the scale problem of small targets in remote sensing images, most existing techniques adopt feature pyramid structures for feature fusion, where only summation or concatenation is used to merge pyramid features. However, various feature pyramid network structures fail to effectively exploit the correlation among all pyramid feature maps, which may lead to a decrease in model accuracy. The SSFF [27] module utilizes a novel scale sequence feature fusion method, which can better integrate the high-dimensional information of deep feature maps with the detailed information of shallow feature maps. During the image downsampling process, the size of the image changes, but the scale-invariant features remain unchanged. Scale space is constructed along the scale axis of the image, representing not only a scale but also a range of scales that an object may have. The scale signifies the details of the image. A blurred image may lose details, but the structural features of the image can be preserved. The SSFF structure is shown in Figure 3. Scaled images, which serve as inputs to SSFF, can be obtained using the following method:

(4) $$F_{\sigma }\left(\omega ,h\right)=G_{\sigma }\left(\omega ,h\right)\times f\left(\omega ,h\right)$$

(5) $$\;G_{\sigma }\left(\omega ,h\right)=\frac{1}{2\pi \sigma^{2}}e^{-\left(\omega^{2}+h^{2}\right)/2\sigma^{2}}$$

Here, $f\left(\omega ,h\right)$ represents a two-dimensional input image with width ω and height h. $F_{\sigma }\left(\omega ,h\right)$ is generated by smoothing through a series of convolutions using a two-dimensional Gaussian filter $G_{\sigma }\left(\omega ,h\right)$, where σ is the scaling parameter of the standard deviation of the Gaussian filter used for convolution. These resulting images have the same resolution but different scales. Therefore, feature maps of different sizes can be considered scale space, and effective feature maps of different resolutions can be adjusted to the same resolution for concatenation. The SSFF module horizontally stacks feature maps of different scales and extracts their scale sequence features using 3D convolution. The SSFF module comprises the following components:

(1) Use a 1 × 1 convolutional layer to change the number of channels in the P4 and P5 feature layers to 256.
(2) Resize them to the size of the P3 layer using the nearest neighbor interpolation method [28].
(3) Increase the dimension of each feature layer using the unsqueeze method, changing them from three-dimensional tensors [height, width, channels] to four-dimensional tensors [depth, height, width, channels].
(4) Concatenate the 4D feature maps along the depth dimension to form a 3D feature map for subsequent convolution.
(5) Finally, accomplish scale sequence feature extraction using 3D convolution, 3D batch normalization, and the SiLU [29] activation function.

### 4.3. HPANet

**Figure 4:**
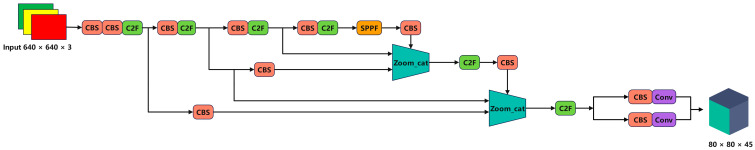
The HPANet network architecture.

In YOLOv8, PANet [30] (Path Aggregation Network) is a network structure designed for multi-scale object detection. PANet aims to address the limitations of single-scale object detection models when dealing with multi-scale objects by effectively aggregating feature maps from different levels to enhance the model’s detection capability. Its advantage lies in its ability to improve the detection accuracy of small objects by extracting and fusing multi-scale features, thus enabling the model to better capture the characteristics of small objects. However, due to the smaller size and fewer features of small objects, the feature fusion and aggregation operations in PANet may lead to the model overly focusing on large objects, thereby reducing the detection performance of small objects. Additionally, the increased computational complexity and parameter volume of PANet also affect the efficiency of handling small objects. Inspired by the PANet network structure, this paper proposes an efficient and lightweight HPANet network structure. In deep learning, the receptive field refers to the perception range of each pixel in the output feature map of a neural network on the input image. The size of the receptive field determines the network’s understanding of local information in the input image, thus affecting the network’s detection capability for objects of different sizes. Therefore, selecting the appropriate receptive field [31] size is crucial for accurate and efficient detection of small objects in remote sensing images. HPANet, based on PANet, removes the detection layers for large and medium-sized objects, thus retaining the detection layers with smaller receptive fields, reducing the computational complexity and parameter volume of the network, and enabling the network to focus on detecting small objects, thereby improving the accuracy and speed of the network. Furthermore, to better utilize information at different scales, enhance the model’s perception of objects, and avoid information loss during feature extraction, the original concatenation fusion method is replaced with Zoomcat after balancing computational complexity and parameters. Zoomcat’s advantages in object detection primarily include multi-scale feature fusion, enhanced perception of multi-scale objects, reduced information loss, flexibility, universality, and improved model performance. By finely fusing feature maps at different levels, Zoomcat effectively captures object information at different scales, thus enabling the model to adapt to various object sizes and handle object detection tasks at different scales within the same model structure. Additionally, the introduction of Zoomcat can avoid information loss during feature fusion and improve the model’s robustness and performance in complex scenes. The HPANet network structure is illustrated in Figure 4.

### 4.4. Modified Model

**Figure 5:**
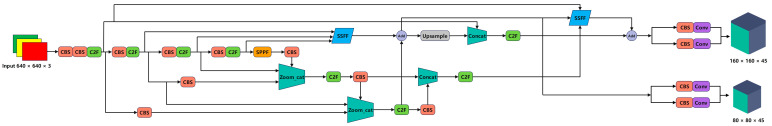
The model architecture after improvement in this paper.

The improved model structure is illustrated in Figure 5 of this paper. This model adopts the HPANet network structure to incorporate the small object detection layer, thus enabling the network to focus on small object detection while reducing parameters and model size. Additionally, the SSFF module is embedded into the neck network, thus allowing the network to better integrate the high-dimensional information of deep feature maps and the detailed information of shallow feature maps, thereby improving detection accuracy.

## 5. Experimentation and Analysis

### 5.1. Data Set and Experimental Environment

**Figure 6:**
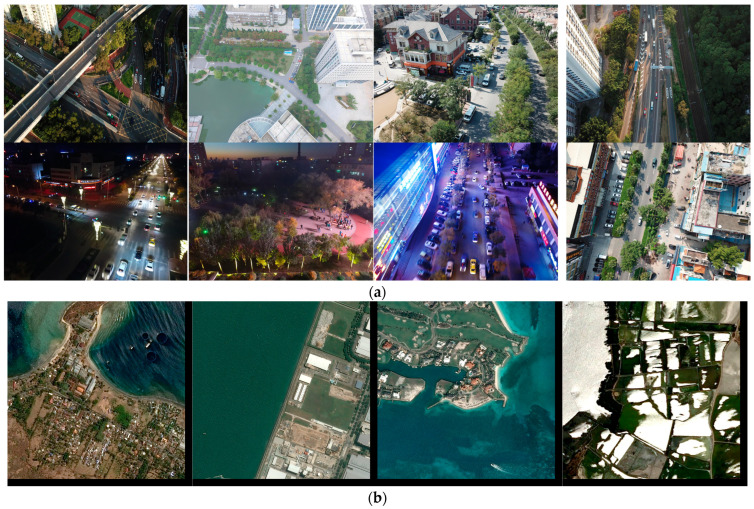
Data sets: (**a**) VisDrone data set; (**b**) AI-TOD data set.

In this thesis, the proposed model is evaluated and validated using the VisDrone UAV aerial image data set, a large-scale data set dedicated to UAV vision tasks [32]. The data set is a large-scale data set for aerial view target detection and tracking, and it is designed to provide rich, realistic vision data for vision algorithm research and development. There are a total of 8599 images, and the training set, validation set, and test set contain 6,471,548, and 1580 images, respectively, with a pixel size of 2000 × 1500, including pedestrians, people, bicycles, automobiles, vans, trucks, tricycles, awning tricycles, buses, and motorcycles, in a total of 10 categories. The VisDrone data set, captured by a variety of UAV-mounted cameras, covers a wide range of aspects, including location, environment, objects, and density. The VisDrone data set has the following characteristics: first, it contains a large number of real scene images covering a wide range of environments, such as cities, villages, and highways, which can well-reflect target detection and tracking scenarios in the real world. Secondly, the labeling information in the data set is very fine, and the location, size, and shape of the target are labeled in detail, which provides an accurate standard for algorithm research. In addition, the VisDrone data set provides images under different weather and lighting conditions, as well as viewpoints under different angles and heights, which can help the algorithms to be applied in various complex situations. As a publicly available data set, the VisDrone data set has been widely used in research and practice in the fields of target detection, tracking, and remote sensing. Researchers can utilize this data set to test and evaluate their algorithms and compare them with other algorithms. At the same time, this data set also provides a valuable resource for industry applications, which can help develop smarter surveillance systems, intelligent transportation systems, and so on. Other data sets of the same type are DOTA [33], AI-TOD [34], etc. Figure 6 shows some images of the VisDrone data set [32] (Figure 6a) and some images of the AI-TOD data set (Figure 6b).

The experiments are based on the PyTorch deep learning framework, and they use a single NVIDIA Tesla T4 graphics card for model training. The specific configuration of the experimental environment is shown in Table 1.

### 5.2. Evaluation Indicators

In target detection, Precision (P), Recall (R), and mAP (mean average precision) are important metrics for evaluating the performance of an algorithm. Precision: precision measures how many of all samples categorized by the model as positive instances are truly positive instances. Recall: the recall rate measures how many of all true positive cases are successfully detected by the model. The specific formulas for P and R are as follows:

(6) $$P=\frac{TP}{TP+FP}$$

(7) $$R=\frac{TP}{TP+FN}$$

The TP in the above formula refers to the number of positive samples correctly classified as positive by the model. FP refers to the number of negative samples incorrectly classified as positive by the model. FN refers to the number of positive samples incorrectly classified as negative by the model. AP is used to measure the performance of the model on a single class. It is the area under the Precision–Recall curve. The formula is as follows:

(8) $$AP=\int_{0}^{1}PRDR$$

The mAP is a commonly used evaluation metric for target detection, which combines the precision and recall of different categories. First, for each category, the area under its Precision–Recall curve (AP, average precision) is calculated, and then the AP of all categories is averaged to obtain the mAP. Its formula is as follows:

(9) $$mAP=\frac{\sum_{k=1}^{n}AP_{k}}{n}$$

### 5.3. Ablation Study

**Figure 7:**
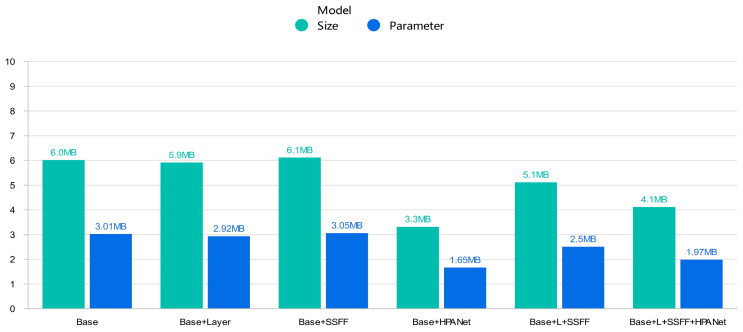
mAP@0.5 and mAP@0.95 data visualization bar graphs from Table 2.

In order to verify the effectiveness of the three improvement points proposed in this paper, we use YOLOv8n as the baseline model for comparison, and we train the VisDrone data set under the same experimental environment. The number of training rounds is 300 epochs, and experiments with input image sizes of 640 × 640 were performed to obtain the test data of each improvement module. The results are shown in Table 2, where “√” indicates that the module is added, and “×” indicates that it is not added. Figure 7 is a visual bar chart of model size and parameter amount in Table 2.

**Figure 8:**
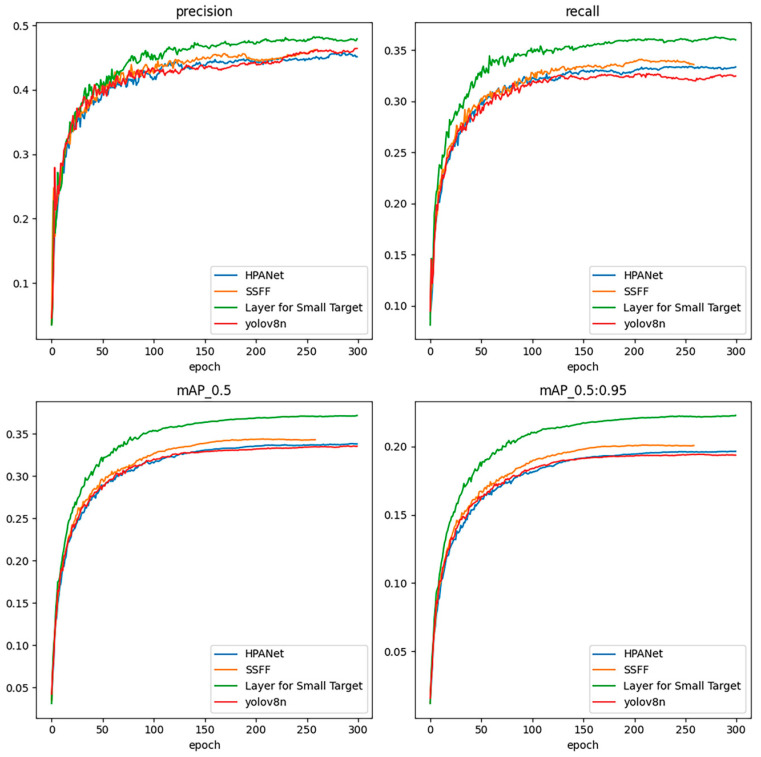
Precision.recall.mAP@0.5 and mAP@0.95 visualization comparison of the three improvement points.

Figure 8 shows the precision, recall, mAP@0.5, and mAP@0.95 curves of YOLOv8n with the addition of HPANet, SSFF, and Layer for Small Target, respectively, and compares them with the base model YOLOv8n, where it can be clearly observed that all three individual improvements are effective.

**Figure 9:**
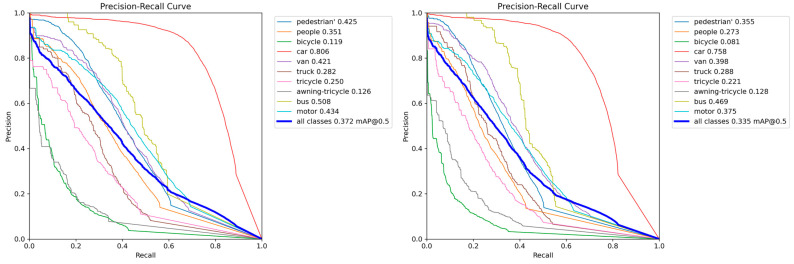
YOLOv8n+Layer for Small Target and YOLOv8n PR curves.

As can be seen from Figure 9, after adding the small target layer alone in YOLOv8, except for Awning-tir accuracy, which is basically the same as that of the base model, all other accuracies are higher than those of the base model, and it can be concluded that the addition of the small target layer is very effective.

Experimental validation has demonstrated that the proposed enhancements in this paper significantly contribute to improving detection accuracy while reducing both model parameter count and size. For VisDrone data set, when introducing the small target detection layer alone into YOLOv8n, there was an 11% increase in mAP@0.5% compared to the original algorithm, with a 15.5% improvement in mAP@0.5:0.95. Introducing the SSFF module alone resulted in a 2.6% improvement in mAP@0.5 accuracy and a 4% improvement in mAP@0.5:0.95. When HPANet was introduced alone, mAP@0.5 and mAP@0.5:0.95 improved by 0.9% and 1.5%, respectively, with a 45% reduction in model size and a 45% reduction in the amount of parameters. Upon introducing both the small target detection layer and the SSFF module simultaneously, the mAP@0.5 increased by 11.6% compared to the original algorithm, with improved detection accuracy observed for various types of small targets. When all three modules were incorporated, our algorithm achieved a 14.3% increase in mAP@0.5 compared to the original YOLOv8n algorithm, with a 17.1% increase in mAP@0.5:0.95, while reducing the model parameter count and size by 33% and 31.7%, respectively. These findings indicate that the enhanced algorithm effectively enhances the model’s capability to extract fine-grained features from remote sensing images, thereby enhancing the overall detection accuracy of the model. In addition, in terms of the model being lightweight, the base model YOLOv8n needs 0.0053s to process an image, and the improved model in this paper needs 0.0079s to process an image, although the processing speed has decreased. However, after our analysis of the growth of model accuracy, the number of parameters and the model storage size are obviously reduced in the trade-off analysis, and we conclude that the improved model in this paper reaches the lightweight standard.

**Figure 10:**
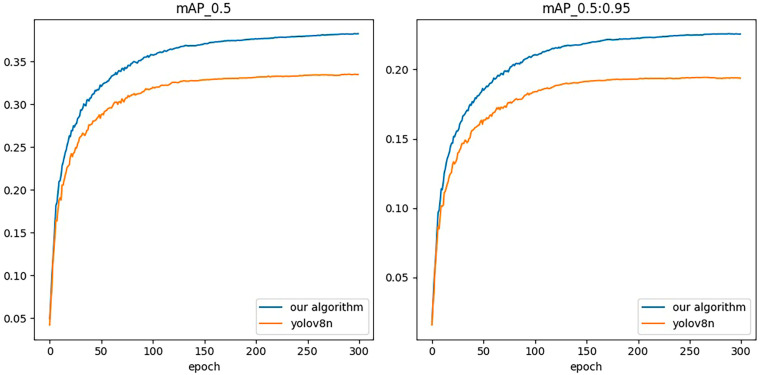
Comparison of mAP@0.5 and mAP@0.95 curves before and after improvement.

The comparison curves of the experimental results of the original algorithm and the improved algorithm on the VisDrone data set are shown in Figure 10. From the figure, it can be seen that the mAP@0.5 and mAP@0.95 of the improved algorithm gradually increase relative to the original YOLOv8 algorithm as the training epoch increases.

**Figure 11:**
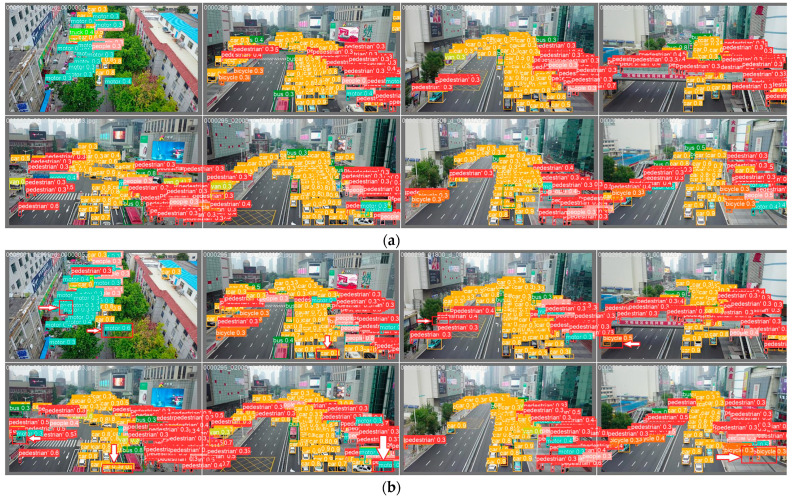
(**a**) YOLOv8n detection visualization results on the data set VisDrone. (**b**) Detection visualization results of the improved model on the data set VisDrone.

Figure 11 shows the visualization results of YOLOv8n and the improved model of this paper on the VisDrone data set for detection, respectively. Based on the comparison, it can be clearly seen that the model proposed in this paper not only has significantly fewer misdetections and omissions but also higher recognition accuracy.

### 5.4. Comparison Test

**Figure 12:**
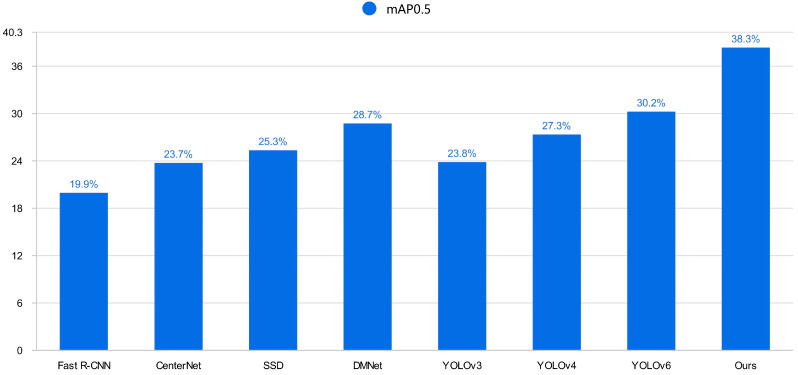
Table 3 mAP@0.5 accuracy visualization bar graphs.

To further validate the effectiveness of the proposed algorithm, various state-of-the-art (SOTA) models were selected for comparison on the VisDrone data set. These include classic YOLO series networks, the anchor-free algorithm CenterNet, the two-stage algorithm Faster-RCNN, and some currently popular algorithms. The experimental results evaluating the improved algorithm based on mAP values for each category and the overall algorithm mAP value are presented in Table 3. In terms of detection accuracy, the improved algorithm in this paper outperforms current popular SOTA models. The overall mAP values of the improved algorithm are increased by 60.9%, 40.2%, and 26.8% compared to the YOLOv3, YOLOv4, and YOLOv6 models, respectively. Moreover, the improved detection algorithm in this paper achieves higher accuracy in each category compared to models of the same category, and the overall model mAP@0.5 reaches 38.2%. These results indicate that the improved algorithm proposed in this paper can effectively accomplish the task of Unmanned Aerial Vehicle (UAV) object detection. Figure 12 is a visual bar chart of mAP0.5 for each algorithm in Table 3.

**Figure 13:**
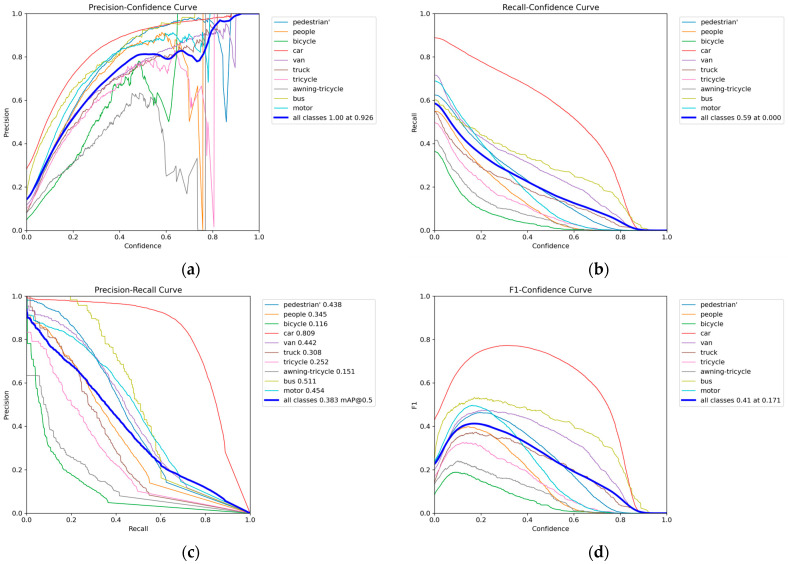
Visualization of evaluation parameters: (**a**) P.curve; (**b**) R.curve; (**c**) PR.curve; (**d**) F1.curve.

Figure 13 presents the visualized evaluation metrics of the improved model proposed in this paper on the VisDrone data set. Figure 13b–d and Figure 13a depict precision, recall, the precision–recall (P-R) curve, and the harmonic mean of precision and recall, respectively. Through analysis, it is evident that the improved model in this paper achieves the best detection precision while maintaining a high recall rate. Compared to the base model, the improved algorithm predicts more accurate results.

### 5.5. Validation on Other Data sets

In order to ensure that the algorithm proposed in this paper can successfully achieve detection in data sets with complex terrain, such as hilly terrain, we have taken several approaches to address the challenges. First, we introduce the SSFF module, which utilizes a new scale-sequence feature fusion approach that can effectively preserve the features of the objects to be detected, thus achieving successful detection. Second, we adopt a multi-scale training strategy, which is able to cover the target features at different scales and improve the generalization ability of the model by using multi-scale image data in the training process. In addition, we adopt various data enhancement strategies, such as random scaling, random cropping, random rotation, etc., to increase the diversity of the training data, which in turn improves the robustness of the model. During the model training process, we performed model optimization and parameter tuning by adjusting the hyperparameters, such as network structure, loss function weights, learning rate, etc., in order to improve the detection accuracy of the model in different scenarios. Finally, after the detection results were output, we performed post-processing optimization, such as adjusting the threshold and parameters of non-maximum suppression (NMS), to further improve the quality of the detection results. In summary, through the combined application of these methods, we are able to effectively address the detection challenges in complex terrain data sets and ensure the performance and accuracy of the algorithms.

In order to validate the robustness and generalizability of the improved algorithm proposed in this paper, this paper chooses to use the AI-TOD [34] aerial image microtarget detection data set, which contains eight different object classes, i.e., airplanes, bridges, storage tanks, ships, swimming pools, automobiles, pedestrians, and windmills, with a total of 700,621 object instances. In order to ensure the comprehensiveness of the evaluation, the data set was divided into a training set and a test set in a ratio of 8:2, where the training set contained 11,214 samples and the test set contained 2804 samples. The largest object in the data set is less than 64 pixels in size, about 86% of the objects in the data set are less than 16 pixels in size, and the average object size is about 12.8 pixels, so the effectiveness of the algorithm proposed in this paper can be fully verified.

**Figure 14:**
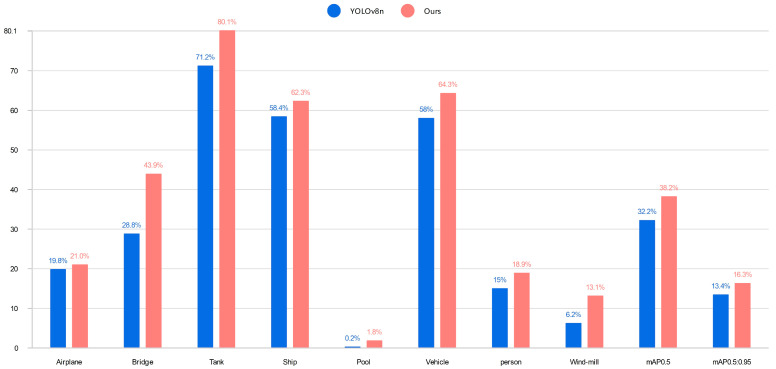
Table 4 data visualization bar chart.

Table 4 shows the results of YOLOv8n and the improved algorithm in this paper on the AI-TOD data set. mAP0.5% and mAP0.5:0.95% of our algorithm on the AI-TOD data set have increased by 18.6% and 21.6%, respectively, which is a significant increase. In addition, there is a significant increase in the accuracy of all categories, of which the bridge and windmill category detection accuracy is much higher than the results obtained by the YOLOv8n model. The improved algorithm in this paper demonstrates good robustness and generalization after the validation of AI-TOD data. Figure 14 is the data visualization bar chart in Table 4.

**Figure 15:**
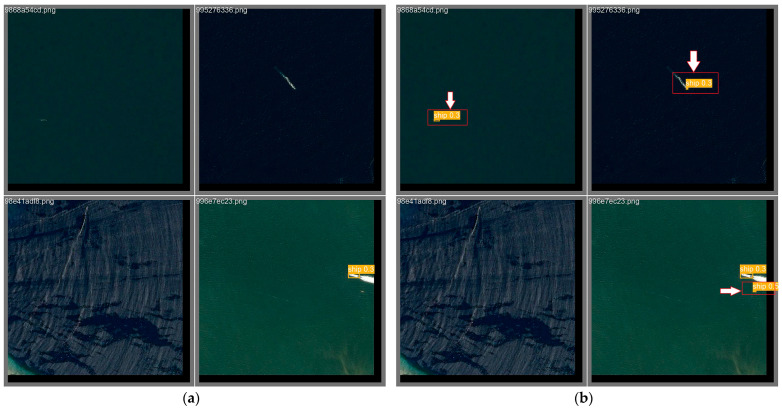
(**a**) YOLOv8n detection visualization results on the data set AI-TOD. (**b**) Detection visualization results of the improved model on the data set AI-TOD. The red box highlights the differences between the left and right pictures.

Figure 15 shows the visualization results of YOLOv8n and the improved model of this paper on the AI-TOD data set, respectively; based on the comparison, it can be seen that the improved algorithm of this paper still has good detection accuracy with the same kind of data set.

## 6. Conclusions

In this study, firstly, we address the challenge of small target detection in remote sensing images by successfully introducing a small target detection layer, which is able to effectively preserve small target features that may be lost during the downsampling process and significantly improve the accuracy of the model. This innovation enables our algorithm to better handle the case of complex backgrounds and dense targets, thus bringing important performance gains to the task of target detection in remote sensing images.

However, after solving the problem of small target feature retention, we found that the existing feature fusion approach is still insufficient, so we further innovated by introducing the SSFF (Scale Sequence Feature Fusion) module into the neck network of the YOLOv8n model. The introduction of this module changes the feature fusion strategy by effectively fusing semantic and spatial information to further improve the accuracy of the model, and it successfully reduces the number of parameters and the overall size of the model. This novel feature fusion mechanism makes our algorithm more adaptable in dealing with challenging situations, such as complex lighting and severe occlusion.

In addition, based on the characteristics of the model data observed in the experiments, we independently developed HPANet and applied it to the model to replace the original PANet, which has a very small model size and fast computing speed, and, at the same time, excellent performance in small target detection accuracy, which is better than PANet in the small target detection task. This innovation not only improves the performance of the algorithm but also further reduces the size of the model, making the algorithm more suitable for deployment and application in real-world scenarios.

We conducted rigorous experiments on two professional remote sensing data sets, VisDrone and AI-TOD, and the experiments proved that compared with the basic YOLOv8n, the improved model accuracy has been greatly improved, the number of parameters and the model size have been significantly reduced to the degree of lightweight, and the algorithm also has a good robustness and generality, and it can effectively cope with a variety of data changes and noise. Comparison tests are conducted on some existing models with high application rates, and the experiments prove that the algorithm proposed in this paper performs well and outperforms the original algorithm and other typical target detection algorithms in terms of accuracy, speed, and model size.

In the future, the algorithmic improvements proposed in this paper can focus on several key aspects. First, the detection accuracy can be improved through more in-depth network structure design and loss function optimization to ensure that the model can accurately capture small target information in remote sensing images. Second, more targeted data enhancement methods need to be designed for remote sensing image characteristics, including simulating image changes under different weather and illumination conditions and more effective broadening of small targets, so as to improve the model’s generalization ability and robustness. In addition, a target feature enhancement mechanism can be introduced to enhance the detection performance by utilizing the target’s own shape, texture, and other feature information, which helps to improve the model’s ability to detect small targets. Meanwhile, the design of a suitable adaptive receptive field mechanism enables the model to dynamically adjust the size of the receptive field according to the size and complexity of the target, so as to detect small targets of different scales more effectively. Considering these aspects of improvement together, the performance and applicability of the algorithm in this paper will be further improved in the task of small target detection in remote sensing images.

## Figures and Tables

**Table 1:** Experimental environment configuration.

Item	Name
Operating system	Ubuntu 20.04
CPU	Intel(R) Xeon(R) Silver 4214R
GPU	NVIDIA Tesla T4
RAM	16 GB
Deep learning framework	PyTorch (2.1.0)
Interpreter	Python (3.8)
CUDA version	CUDA (11.8)

**Table 2:** Ablation study results.

Base	Layer for Small Target	SSFF	HPANet	mAP_0.5_	mAP_0.5:0.95_	FPS	Model Size/MB	GFLOPs	Parameter/MB
√	×	×	×	33.5	19.3	187.8	6.0	8.1	3.01
√	√	×	×	37.2	22.3	128.6	5.9	12.2	2.92
√	×	√	×	34.4	20.1	153.9	6.1	8.5	3.05
√	×	×	√	33.8	19.6	293.3	3.3	6.3	1.65
√	√	√	×	37.4	22.1	107.1	5.1	12	2.5
√	√	√	√	38.3	22.6	125.3	4.1	13.6	1.97

**Table 3:** Detection results of different algorithms on VisDrone data set.

Algorithm Model	Awn-tr	Bicycle	Bus	Car	Motor	Pedestrian	People	Truck	Tricycle	Van	mAP_0.5_
Fast R-CNN	8.73	5.8	43.8	44.1	16.8	12.5	8.1	30.4	8.5	20.4	19.9
CenterNet [35]	14.2	7.5	42.6	61.9	18.8	22.9	11.6	24.7	13.1	19.4	23.7
SSD	11.1	7.3	49.8	63.2	19.1	18.7	9.0	33.1	11.7	29.9	25.3
DMNet [36]	14.1	8.9	49.2	58.9	29.3	27.6	18.9	29.3	20.3	30.2	28.7
YOLOv3	7.7	6.8	39.3	68.8	21.5	22.5	12.5	26.4	8.4	24.3	23.8
YOLOv4	12.3	8.6	48.8	69.2	22.7	26.6	14.5	29.9	12.6	27.2	27.3
YOLOv6	10.2	5.0	43.0	74.1	32.4	31.4	25.5	26.7	18.1	35.7	30.2
Ours	15.1	11.6	51.1	80.9	45.4	43.8	34.5	30.8	25.2	44.2	38.3

**Table 4:** Results of YOLOv8n and the improved model on the AI-TOD data set.

Algorithm Model	Airplane	Bridge	Tank	Ship	Pool	Vehicle	Person	Windmill	mAP_0.5_	mAP_0.5:0.95_
YOLOv8n	19.8	28.8	71.2	58.4	0.2	58.0	15.0	6.2	32.2	13.4
Ours	21.0	43.9	80.1	62.3	1.8	64.3	18.9	13.1	38.2	16.3

## Data Availability

Data openly available in a public repository. The VisDrone data supporting the results of this study are publicly available at [https://github.com/VisDrone/VisDrone-Dataset] (acceded on 1 March 2024). The AI-TOD data supporting the results of this study are publicly available at [https://github.com/jwwangchn/AI-TOD] (acceded on 1 March 2024).
